# At the frontier of epigenetics of brain sex differences

**DOI:** 10.3389/fnbeh.2015.00221

**Published:** 2015-08-21

**Authors:** Margaret M. McCarthy, Bridget M. Nugent

**Affiliations:** ^1^Department of Pharmacology, University of Maryland School of MedicineBaltimore, MD, USA; ^2^Department of Biomedical Sciences, School of Veterinary Medicine, University of PennsylvaniaPhiladelphia, PA, USA

**Keywords:** estrogens, preoptic area, amygdala, reproductive behavior

## Abstract

The notion that epigenetics may play an important role in the establishment and maintenance of sex differences in the brain has garnered great enthusiasm but the reality in terms of actual advances has been slow. Two general approaches include the comparison of a particular epigenetic mark in males vs. females and the inhibition of key epigenetic enzymes or co-factors to determine if this eliminates a particular sex difference in brain or behavior. The majority of emphasis has been on candidate genes such as steroid receptors. Only recently have more generalized survey type approaches been achieved and these promise to open new vistas and accelerate discovery of important roles for DNA methylation, histone modification, genomic imprinting and microRNAs (miRs). Technical challenges abound and, while not unique to this field, will require novel thinking and new approaches by behavioral neuroendocrinologists.

In 2009 a minisymposium was held at the annual meeting of the Society for Neuroscience titled “Epigenetics of Sex Differences in the Brain”. Representatives from five laboratories presented their findings to a standing-room-only audience, indicative of the burgeoning interest in both epigenetics in the nervous system and the origins and maintenance of sex differences in the brain. But the interest far out distanced the field. The concluding remarks at the symposium summarized that what we had just listened to was largely made up of promises, promises of future progress with very little hard data actually in hand (McCarthy et al., 2009a). Some progress has been made in the intervening 5 plus years, but given the general rate at which research moves in the modern era, the progress is surprisingly little. Why would this be? A number of factors can be blamed for slowing the pace. First is simply the small number of labs that work on the topic of epigenetics and sex differences. This may change in the coming years with the implementation of new policies by the National Institutes of Health requiring equal representation of males and females in preclinical research (Clayton and Collins, 2014), but then again it may not. Second is the vastness of the topic. Epigenetics is increasingly a general term that encompasses many and varied specifics. These include changes to the DNA via methylation, which become more and more complicated the more we learn, changes to the histones of which there is not quite an infinite variety but something close, imprinting, a still mysterious process of allelic regulation especially in the brain, and microRNAs (miRs) which can impact multiple genes simultaneously. Then there are the technical challenges, not the least of which is the need for bioinformatics expertise to handle the voluminous data generated when conducting sequencing experiments. Together these factors create a substantial energy barrier to the pursuit of epigenetic analyses, nonetheless interest in the topic remains high.

Why epigenetics is important to sex differences is embedded in several lines of thought. From a strictly biological standpoint one could argue that epigenetic regulation provides for the assurance that gonadal sex and brain sex are coordinated independent of genetic sex, i.e., chromosome complement. From an evolutionary standpoint the goal is procreation and thus an animal’s behavior needs to be coordinated with gamete production and fertilization, regardless of genetic constitution. By allowing for epigenetic regulation of behavior, driven by gonad-derived signals such as steroids, this coordination can be assured. Moreover, some species exhibit facultative sex changing and the placing and removing of epigenetic marks is a simple way to achieve that end. But epigenetic regulation of sex differences is also important to influences not considered directly biological, such as experience or environment in animals and culture and society in humans. Repeated sex-typic experiences or expectations could feasibly impact the epigenome in a manner that further enhances or even canalizes sex differences. Conversely, the malleability of epigenetics could contribute to discordance between biological sex and sexually differentiated endpoints such as gender identity or to naturally occurring variations in partner preference. Thus understanding the factors that regulate epigenetic changes in males and females and the impact these changes have on brain and behavior has wide ranging implications and potential impacts.

In addition to the variety of possible epigenetic changes noted above, there is also a need to separate and interpret findings by developmental state, sex (obviously), brain region and cell type. When considering brain sex differences all of these are fundamental, beginning with developmental stage. The principle drivers of sex differences are steroid hormones, which differ profoundly in males and females at some times of life and very little or not at all at other times of life. Early exposure to steroids has enduring effects and most brain sex differences are established during a critical developmental window, although the timing of the window may vary for different endpoints as well as for masculinization vs. feminization. For steroid-mediated masculinization of reproductive behavior, the beginning of the critical window is delineated by the onset of gonadal steroidogenesis by the testis of the fetal male (around embryonic day 18 in rats and mice) and the close is the developmental age at which a female brain is no longer sensitive to exogenously induced masculinization which is accomplished by steroid injection (Figure 1). In our rodent models this is about 1 week after birth. Thus the sensitive period is perinatal, i.e., both prior to and just after birth. In primates evidence to date suggests the process of brain sexual differentiation is largely prenatal although the exact parameters are difficult to firmly establish for the obvious reasons associated with work in humans and primates. The interested reader is referred to the following reviews for a more thorough discussion of the details of sexual differentiation (Arnold et al., 2003; De Vries, 2004; Morris et al., 2004; Forger, 2006; de Vries and Södersten, 2009; McCarthy et al., 2009b, 2012; McCarthy and Arnold, 2011; Shen et al., 2015).

**Figure 1:**
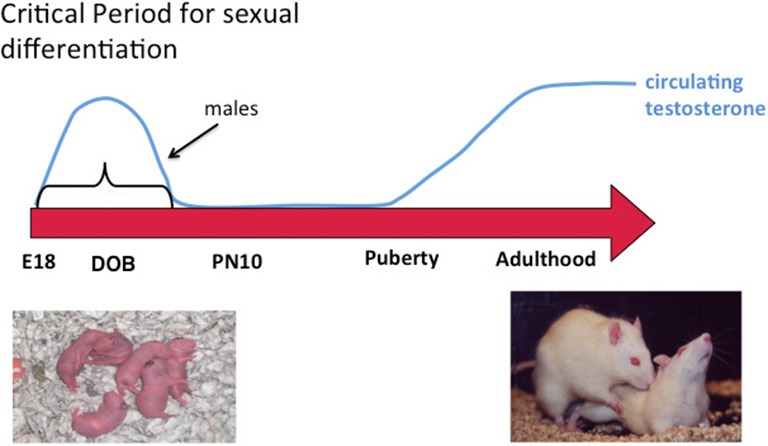
**Sexual differentiation of brain and behavior.** Sex differences in the brain are established early in development during a critical period. Feminization of the brain proceeds in the absence of exposure to elevated gonadal steroids during the critical period and masculinization occurs when the fetal testis begins production of androgens at the beginning of the critical period, the end of which is defined by the developmental stage at which exogenous administration of androgens to females is ineffective at switching brain development from feminization to masculinization. Gonadal hormones rise again in adulthood and promote sex differences in behavior by acting on a neural substrate that was organized differently in males and females. A central question in behavioral neuroendocrinology has been how early life exposure to androgens exerts an enduring influence on adult brain and behavior.

During the critical period circulating testosterone is markedly higher in males and some portion of this gains access to the brain where it is locally converted into estradiol. Maternal estradiol also gains access to the fetal circulation but much of it is sequestered there by the steroid binding globulin alpha-fetoprotein. There is detectable estradiol in the brains of males and females during the critical period and it varies substantially across brain region. Despite this, the variation in levels between males and females is found only in a few brain regions, mostly the preoptic area (POA), and this sex difference is small in magnitude and brief in duration, being nonexistent within hours after birth (Amateau et al., 2004; Konkle and McCarthy, 2011). Nonetheless, in rodents estradiol is the dominant driver of most sex differences in brain and behavior. Of additional interest, effective masculinization of female pups with exogenous estradiol injection requires an enormous dose, ten times that used in a full grown adult to induce sexual receptivity. This rather bizarre scenario raises some interesting questions; how does such a small and transient sex difference in steroid levels have such a profound effect? Why is it so hard to masculinize females with exogenous steroid? One possibility is that forces within the genome act to maintain femaleness and prevent maleness. One of the likely forces would be at the epigenetic level and this potential is discussed further below.

There are two general approaches to the study of brain sex differences and epigenetics. The first is to simply measure epigenetic marks in males and females and compare them. The second is to take a known endpoint that is different in males and females, be it morphological or behavioral, and determine if manipulating the system, by blocking or stimulating a key enzyme for instance, disrupts or mimics the formation of the sex differences (Figure 2). Each approach has proven fruitful in its own way. Several recent reviews have effectively cataloged what we currently know and this can be largely summarized as there are sex differences in multiple brain regions in multiple epigenetic marks (Nugent et al., 2010; Nugent and McCarthy, 2011; Auger and Auger, 2013; Kigar and Auger, 2013; Matsuda, 2014). One of the major principles emerging in the larger field of epigenetics is the integral relationship between DNA methylation and histone modifications (Cheng, 2014). Put simply, any change in DNA methylation is likely to be accompanied by some change to the associated histones, and* vice versa*. But for most investigators analysis is either at the histone or DNA level, unless the work specifically targets this interaction. Yet there is good reason to focus on both, as both types of modifications have been found to either be important for the process of sexual differentiation or changed in response to sexual differentiation. Here are some specifics.

**Figure 2:**
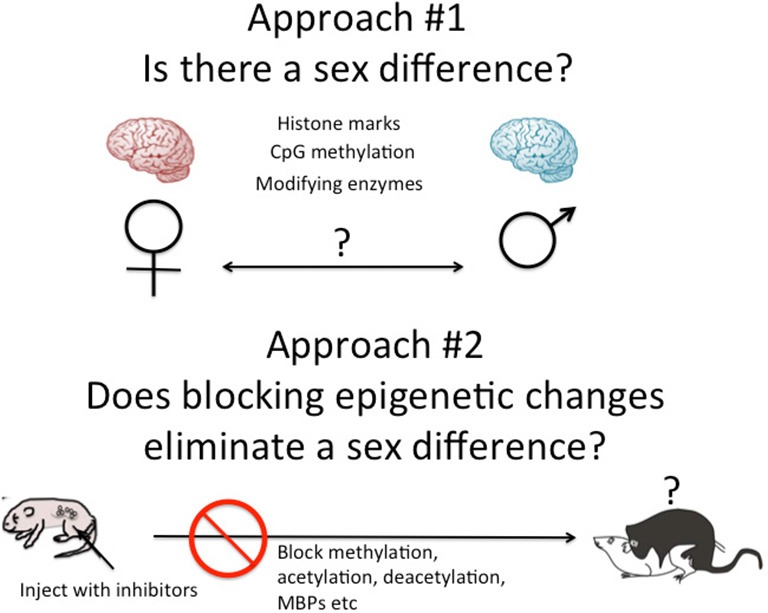
**Two approaches to epigenetics of brain sex differences.** One approach is to simply measure known epigenetic marks such as histone acetylation or CpG methylation, and/or the enzymes known to regulate the establishment and maintenance of these marks and ask, are they different in males and females? A second approach is to disrupt the establishment of epigenetic marks early in development by inhibiting the associated enzymes or cofactors such as methyl binding proteins (MBPs) and ask, does this eliminate sex differences in adult brain and behavior?

## Brain Sex Differences in Histone Modifications

The best studied form of histone modification is acetylation of lysines on the extruding polyamine tails, mostly H3 but also H4 and H2A. Additional modifications at some of the same critical lysines can toggle the impact of the chromatin between repressive and permissive (Bernstein et al., 2006; Ku et al., 2013). Individual site changes often don’t operate in isolation but are instead coordinated with and influenced by what is happening at neighboring amino acids, and collectively the changes on the histones at a particular site will ultimately determine gene expression, a phenomenon referred to as the “histone code” (Strahl and Allis, 2000). Antibodies to specific histone modifications allow for crude quantification by Western blot and the use of Chip-Seq is valuable for pulling down and sequencing stretches of DNA associated with a particular modification. The former approach was used by Rissman and colleagues to document a sex difference in the amount of H4 acetylation in the cortex and hippocampus of rat pups (Tsai et al., 2009). Acetylation and deacetylation are separate reactions controlled by distinct enzymes, the histone acetyl transferases (HATs) and the histone deacetylases (HDACs), respectively, both of which have multiple isoforms from large complex families (Lau et al., 2000; Wang et al., 2009; Hayakawa and Nakayama, 2011). Either stimulation or disruption of these enzymes by pharmacologic or gene silencing techniques have been found to impact neuroanatomy (Murray et al., 2009) and sexual differentiation of behavior (Matsuda et al., 2011). In both cases the investigators have focused on their particular system, emphasizing one brain region and one endpoint, either neuroanatomical or behavioral. Inhibiting HDACs with valproic acid on the day of birth increased H3 acetylation within a day, demonstrating that deacetylation is a constitutively active process in this brain region, and this same treatment reduced the size of the principle nucleus of the bed nucleus of the stria terminalis (pBNST). This nucleus is normally larger in males due to greater cell death in this region in females. There was no change in the size of the nucleus in females (Murray et al., 2009), which even though it is normally smaller than in males is nonetheless capable of getting smaller, in fact why didn’t it disappear all together? Something seems to have prevented that from occurring. Importantly the effects of valproic acid treatment were not generalizable to other regions as there was no change in the size of the suprachiasmatic nucleus (SCN) or a thalamic nucleus (Murray et al., 2009). One could speculate that there is a specific gene or gene network that promotes cell survival in the pBNST of males (or, promotes cell death in females) and that it is suppressed via increased acetylation following valproic acid treatment, and hence the cells die, but not all of them, only those that make the nucleus larger in males than in females. Likewise this gene must NOT be important to cell survival in females. This would require there to be two populations of cells in the pBNST, one that is influenced by steroids to survive and regulated by histone acetylation, and one that is not. While possible, this seems unlikely.

Matsuda et al. (2011) took a slightly different approach and explored the epigenetic regulation of genes already known to be central to the process of masculinization of sexual behavior: estrogen receptor (Esr1), or estrogen receptor alpha (ERα) and Cyp19a or aromatase, the enzyme that converts testosterone to estradiol. They found that during the sensitive period for differentiation, HDACs 2 and 4 were more prevalently bound at the promoters of Esr1 and Cyp19a in males than females, which presumably leads to greater deacetylation and higher gene expression. Reduction in HDAC activity with either pharmacological treatment (trichostatin A) or antisense oligonucleotides directed against the mRNA for HDACs 2 and 4, impaired behavioral masculinization.

Both studies are important but could be critiqued for telling us what we largely already knew. What is lacking is exploration and discovery of new sources of brain regulation. Forger and colleagues took an important step in this direction with a genome wide analysis of a single but essential epigenetic mark, H3K4me3, which is permissive of gene expression (Shen et al., 2015). They explored the pBNST in combination with the POA in adult mice and found ~250 genes in which there was a sex difference in associated H3K4me3. The genes identified were associated with synaptic function and while many are not expressed differently at baseline, this does not negate the importance of epigenetic regulation as H3K4me3 has been proposed as a component of bivalent chromatin, meaning it is in a state of readiness for rapid shifts into expression.

These few studies summarize the current state-of-the art in chromatin modifications as a component of sexual differentiation. Somewhat greater progress has been made in the arena of changes to the DNA.

## Brain Sex Differences in DNA Modifications

The canonical epigenetic modulation to DNA is addition of a methyl group to the 5′ carbon of cytosines that are adjacent to guanines, referred to as mCpG. Methylation of cytosines adjacent to other nucleotides is emerging as a particularly important regulator of gene expression in the brain, but is found at very low levels in the immature brain when sex differences are established (Lister et al., 2013). Interest in sex differences in DNA methylation was piqued by the remarkable studies from the Meaney laboratory demonstrating how early life experience, in particular the type of maternal care one receives, impacts the methylation of specific cytosine residues in the promoter of the gene for the glucocorticoid receptor (GR) in the hippocampus and that this, in ways not fully understood, contributes to a transgenerational stability in the intensity of maternal care shown by females (Champagne, 2008; Curley et al., 2011; Kundakovic and Champagne, 2015). These studies highlighted both the importance of steroid receptors and their malleability at the epigenetic level. Champagne and Curley (2008) furthered the field by characterizing epigenetic changes in the promoter of the estrogen receptor (Esr1) in response to hormonal and environmental cues, but they did not address sex differences nor the process of sexual differentiation. Toward this end, Auger and colleagues mapped a region of the Esr1, finding that males had higher levels of methylation than females in tissue from the POA. They further found that the higher methylation in males correlated with reduced ER protein. Moreover, if they stimulated the maternal grooming that had been shown by Meaney and Champagne to impact GR methylation, it also altered Esr1 methylation, only in their case they also mimicked the naturally occurring variation in which the maternal dam grooms her male pups much more frequently than her female pups (Edelmann and Auger, 2011). This behavioral change was determined to underlie the sex difference, thus nicely demonstrating how experience can alter the epigenome and how in turn the epigenome can impact hormone sensitivity of a particular brain region.

Because of the centrality of estradiol to rodent brain sexual differentiation, both isoforms of the estrogen receptor were obvious candidate genes for epigenetic modifications. In an exhaustive study of three brain regions (POA, hypothalamus and hippocampus), three developmental time points (newborn, adolescent and adult), three candidate genes (Esr1, Esr2 and PR) and three groups (males, females and masculinized females) the amount of methylation within CpG islands of the promoters of each gene was found to increase across development and also show transient sex differences, but, none of these changes were permanent nor were they predictive of expression (Schwarz et al., 2010). This highlights much of the challenge with studies of DNA methylation, that the relationship between methylation status and expression is not necessarily a repressive one and that methylation status changes in the brain with age. Moreover, in addition to the methylation itself, DNA methyl binding proteins (MBPs) are important partners in gene regulation. The best known of these is MeCP2, a DNA MBP mutated in Rett Syndrome (Amir et al., 1999). Under normal conditions, MeCP2 is recruited to methylated promoters and further enhances repression of gene expression. Auger and colleagues have exploited the ease of quantifying methyl CpG binding protein 2 (MeCP2) and of inhibiting it, to demonstrate important sex differences and regulation of the sexual differentiation of juvenile play behavior, a social behavior that is expressed at higher levels by males and is importantly controlled by the amygdala (Auger and Olesen, 2009; Auger et al., 2011). Vasopressin is a neuropeptide expressed at higher levels in the male amygdala and MeCP2 represses its expression in females. Emancipating this gene by silencing MeCP2 for even a brief period during development permanently alters the pattern of expression and eliminates the sex difference both in vasopressin levels and in social play behavior (Forbes-Lorman et al., 2012). More recently, they determined that one of the genes regulated by MeCP2 is GFAP, an astrocyte-specific structural protein (Forbes-Lorman et al., 2014), illustrating yet another important point, epigenetics impacts all cell types, a distinction we often miss in our brain region homogenates.

The candidate gene approach can be both useful and fruitless, sometimes telling us what we already know and in other instances not agreeing with what we think we know. For this reason we also need discovery based approaches. What is being epigenetically regulated that we haven’t considered? This type of hypothesis-free approach has the potential to be enormously powerful, but it is also enormously challenging. The genome is large but becomes vastly larger when viewed in the context of DNA methylation. This is in part because much of the methylation occurs outside of genes, in intergenic regions, and how that methylation impacts neural function remains largely unexplored. Add in non-CpG methylation (Guo et al., 2014) and hydroxymethylation, which some call the 6th base (Irwin et al., 2014; Feng et al., 2015) and the complexity level rises still further. Combine this with the use of bisulfite conversion, which is required to detect CpG methylation but is a process that can be overdone or underdone, plus the cost of sequencing, and the energy barrier rises still higher. Finally, if one does get that far, there is the issue of bioinformatics analyses and it is this step that thwarts most labs whose primary focus is on sex differences in the brain and therefore lacking in the skills needed to analyze and understand the millions of reads produced in modern day sequencing facilities. Vilain and colleagues (Ngun et al., 2014) solved the problem nicely with the use of Reduced Representation Bisulfite Sequencing (RRBS) which provides genome wide scanning but is enriched for areas with high CpG content, thereby greatly reducing the amount of sequencing required. As a result the investigators were able to assess the methylation profile in two brain regions (POA and striatum) of males, females and masculinized females at two developmental time points, as neonates and as adults. This is perhaps one of the most comprehensive views of epigenetic sex differences in the brain to-date. One of the most surprising discoveries was how few genes exhibited differential methylation as neonate as opposed to the large number that emerged in adulthood. Moreover, the impact of neonatal testosterone endured, or was revealed, in adulthood, and this was not dependent on adult hormonal status as the gonads were removed at the time of sacrifice. They found over 1,000 genes that were differentially methylated in the striatum of males and females, and over 600 in the adult POA. The delayed emergence in gene methylation differences following neonatal testosterone is a surprising and currently unexplained observation. In contrast to the hundreds of genes that differed in adult brain, the number of sex differential genes in the neonate was closer to 50. Why there is such a divergence in the sex-typic expression profiles between neonates and adults and how the effect of testosterone on gene methylation is delayed until adulthood and maintained independent of circulating hormones is a fascinating and currently unresolved mystery. No doubt the authors will continue to mine this rich database and reveal even more surprises.

## DNA Methylation Represses Masculinization of the POA and Reproductive Behavior

**Figure 3:**
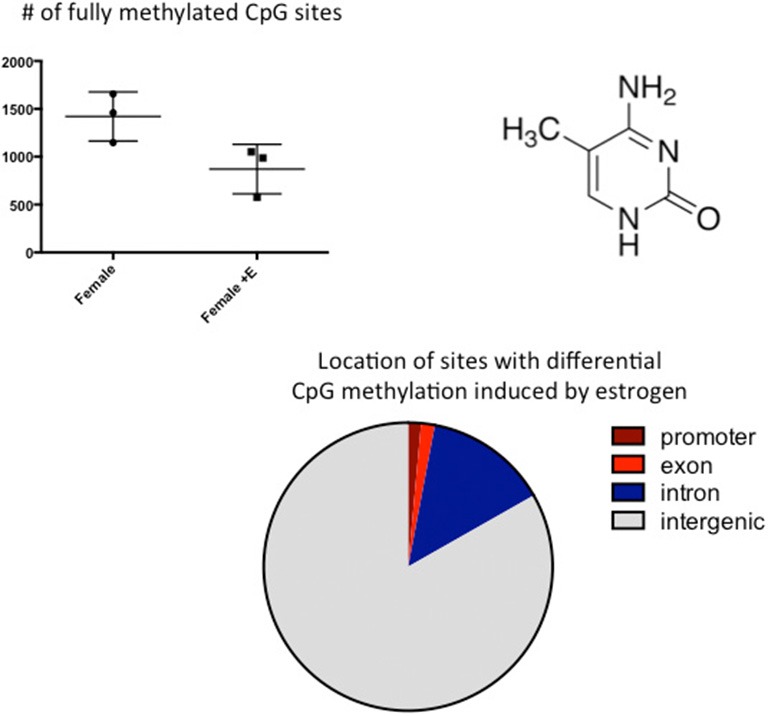
**CpG methylation is modulated by hormones.** Cytosines are methylated at the 5′ carbon by a class of enzymes called DNA Methyl Transferases (DNMTs). The number of fully methylated CpGs was quantified in DNA extracted from the preoptic area (POA) of 3 newborn females and 3 females treated with a masculinizing dose of estradiol for the previous 2 days. Estradiol treatment significantly reduced the number of fully methylated sites and analyses of where in the genome these sex differences reside indicated they are predominantly in the intragenic region, where DNA methylation is highest.

**Figure 4:**
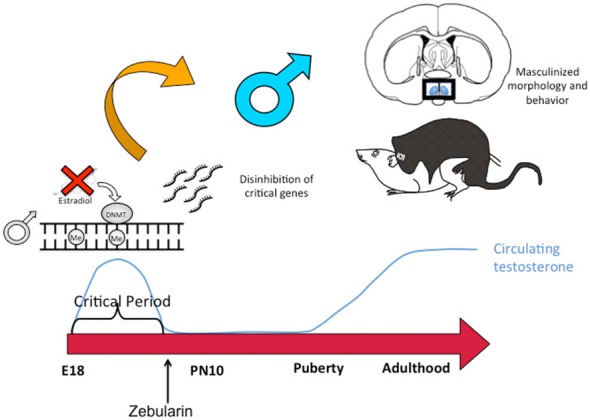
**DNA de-methylation mediates masculinization of brain and behavior.** Treatment of newborn male rat pups with DNMT inhibitors such as Zebularine during the critical period of sexual differentiation reduces DNA methylation and thereby changes gene expression profiles which in turn leads to masculinization of the synaptic patterning of the POA and sexual behavior. Thus masculization requires demethylation while feminization is a repression of masculinization via DNA methylation.

Similar to the discovery based approaches described above, we also took a “big picture” view of the epigenome and sexual differentiation by quantifying the overall level of DNA methylation in the POA of male, female and masculinized female rat pups (Nugent et al., 2015). We found females had significantly higher overall DNA methylation compared to either males or masculinized females. Whole-genome-bisulfite-sequencing (WGBS) revealed this was due to more highly methylated CpG sites in females and that most of this was not in CpG islands or gene promoters but was instead in the intergenic regions, where most DNA methylation is found. Moreover, the sex difference in methylation was broadly distributed across the chromosomes (Figure 3). In order to establish if the sex difference in DNA methylation was functionally important we used two approaches. First was to determine if reducing the DNA methylation in females during the critical window would masculinize the POA and adult reproductive behavior, and the second was to identify what genes were being repressed in females as a result of elevated DNA methylation. Both aims were achieved with the use of DNMT inhibitors, specifically zebularine and RG108, which have slightly different mechanisms but are similar in that they block all DNMT activity and result in de-methylation of the DNA (Yoo et al., 2004). After confirming this was also true in the developing POA, newborn males and females were treated with either RG108 or zebularine, raised to adulthood and tested for male sexual behavior by being placed with a sexually receptive females (all test subjects also underwent gonadectomy and testosterone replacement to both simulate adult male circulating hormone levels and equalize hormone levels across animals). Both the brain and behavior of females treated with DNMT inhibitor as neonates were fully masculinized in adulthood, meaning they exhibited a male-like synaptic pattern and showed normal male sexual behavior (Figure 4). This was not true of females treated with vehicle as neonates, they showed very little male-like behavior despite having equal circulating testosterone levels as the time of testing as the other animals. Thus the demethylation that had occurred some 60 days prior resulted in a gene expression pattern that masculinized the brain. What those genes might be was assessed by conducting RNA-Seq on mRNA from the POA of males and females with and without DNMT inhibitor treatment. To our surprise the number of genes expressed at significantly different levels in male and female POA was relatively small, ~70, but consistent with the low number observed by Vilain and colleagues using RRBS (Ghahramani et al., 2014). About half of the genes were expressed at higher levels in males and half at higher levels in females. Candidate genes for masculinization are those that increased in females following DNMT inhibition and were also higher in control males than females. Of the 34 genes expressed at higher levels in males, 25 of them increased in females treated with a DNMT inhibitor (Nugent et al., 2015). It is logical to predict that in females these genes would have increased DNA methylation in the promoter region compared to males or masculinized females, but exhaustive sequencing analysis found no evidence that was the case, suggesting the change in expression of these genes was secondary to other events or are located outside of promoters.

## DNA Methylation Closes the Sensitive Window for Sexual Differentiation

**Figure 5:**
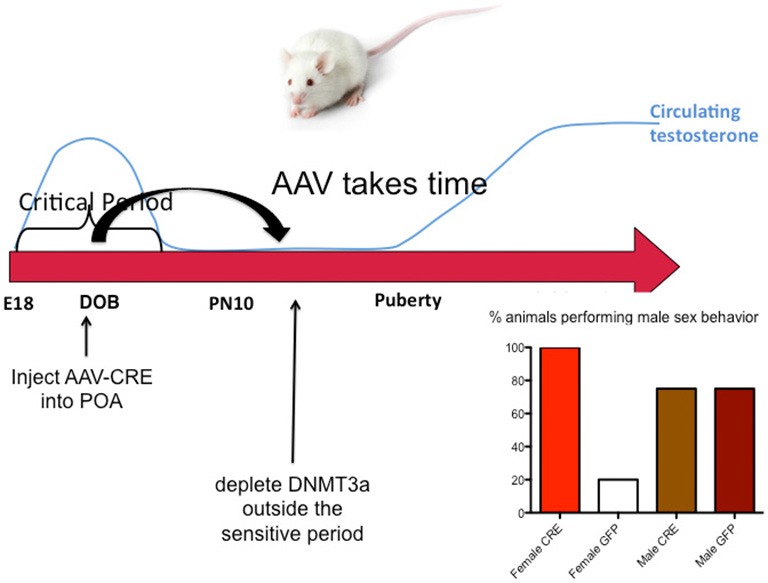
**DNA methylation maintains feminization of brain and behavior.** Conditional knockout of DNMT3a in the POA of mice demonstrated that reduced DNA methylation outside the critical period could still lead to masculinization of behavior, while treatment with exogenous estradiol could not. This was also found to be true in rats using DNMT inhibitors. During the critical period estradiol reduces DNMT activity but this effect is lost outside of the critical period. Thus the ongoing maintenance of DNA methylation appears essential for continued feminization. These observations reveal a novel source of plasticity in sexually dimorphic behavior.

The end of the sensitive period for sexual differentiation is defined as the developmental age at which exogenous hormone treatment is no longer capable of masculinizing females. But why females lose sensitivity to the masculinizing effects of steroids was unknown. We mapped the level of DNMT activity in the POA of males and females from birth to 2 weeks of age and determined that females had significantly higher enzymatic activity during the first few days after birth but that by 4 days of age levels had equalized and by 1 week levels dropped precipitously in both sexes. Thus it appears the first few days of life are highly dynamic for DNMT activity in the POA. Further, treating neonatal females with a masculinizing dose of estradiol reduced DNMT activity to male-like levels, suggesting that at least one mechanism for estradiol mediated masculinization of the POA is via control of DNMT activity. In order to determine if DNA methylation past the sensitive period was required to maintain feminization of brain and behavior, we treated 10-day-old females with DNMT inhibitor and raised them to adulthood. We also treated some females with a masculinizing dose of estradiol at the same time and found that as adults, these females were not masculinized, (i.e., they were outside the sensitive period at this time) but their littermates that had been treated with DNMT inhibitor were masculinized. We observed a similar phenomenon in mice in which the DNMT3a gene was conditionally ablated in the developing POA by an adeno-associated virus (AAV)-Cre mediated knockout. All female DNMT3a floxed mice that received AAV-Cre displayed male sexual behaviors compared to very few control females. However, knockout did not occur until outside the sensitive period (Figure 5). Collectively these data indicate that the end of the sensitive period is a function of the loss of inhibition of DNMT activity by estradiol. How this occurs, from a mechanistic viewpoint, is unknown, as is why DNMT activity drops so markedly at the end of the first week of life. There is much still to be learned regarding the DNA methylome and sexual differentiation of brain and behavior.

## MicroRNAs as Epigenetic Regulators of Brain Sex Differences

miRs are a type of non-coding RNA and now recognized to be ubiquitous influencers of gene expression (Jia et al., 2013). Largely by actively degrading mRNA but also via steric hindrance, these small endogenously synthesized regulators can modulate multiple genes simultaneously to dampen networks of gene expression. Bale and colleagues surveyed 250 miRs in whole neonatal mouse brain and found almost 2/3rds were expressed at different levels in males and females (Morgan and Bale, 2012). This is surprisingly high given they surveyed whole brains and suggests there is a global regulation of gene expression that differs by sex. Of the miRs that differed by sex, half of them were due to gonadal steroid regulation and a third were due to bias in the sex chromosomes. The remainder of the sex differences are of unknown origin. The relative contribution of different cell types, i.e., neurons vs. astrocytes vs. non-neuronal cells is entirely unexplored and may be trivial or, may be pivotal. We have explored miRs in the dentate gyrus of the neonatal hippocampus, providing some regional as well as cellular specificity given the high concentration of granule cells here, and we also found a startling high number of them showed a difference in expression based on sex. Of 14 miRs selected for their role in cell genesis and neural differentiation, ten were found to be higher in females than males (McCarthy et al., 2015). In this case, however, we found no regulation by gonadal steroids and most of these miRs are not associated with the X chromosome. These preliminary findings point to possible regional differences in miR regulation and impact, although species difference cannot be ruled out as well since of the two studies done to-date looking for sex differences in miRs in the brain, one was in mice (Bale) and one was in rats (ours). This will be an exciting area for future exploration.

## Conclusion

With a solid 5 years since what could conceptually be viewed as the beginning of the field of epigenetics of sex differences in the brain its seems we are still at the very edge of the frontier. Genes known for decades to be central to the process of differentiating male from female brains are clearly stamped with epigenetic marks that presumably impact their role in the process. Discovery of new gene participants has been slower to come as we grapple with the biological challenges of cell and regional specificity, the technical challenges of bioinformatics and the interpretive challenges as to what do specific epigenetic marks really tell us. Discriminating between what are genuine epigenetic (although not heritable) modifications that will permanently alter the trajectory and sensitivity of a particular cellular endpoint and attendant behavioral profile from a simple reflection of on going changes in gene expression is an additional challenge. This last challenge is exacerbated by the inability to assay changes in real time in order to gain a more nuanced view of changes above the genome. This is in marked contrast to the traditional physiological approaches of sequential and repeated sampling of the same animal over time so that one could, for instance, create an accurate profile of testosterone changes over the course of a day, a month, a year. Likewise for behavior, does sexual receptivity track with time of day? Year? Hormonal profile? What we would like to know as well is does the epigenetic marks on the genes controlling sexual receptivity also change across the day, month or year but current technology does not allow for repeated sampling of the same individuals brain epigenome. This means we need to think in new ways and frame our questions differently than before in order to illuminate the powerful role of epigenetics in controlling sex differences in brain and behavior.

## Conflict of Interest Statement

The authors declare that the research was conducted in the absence of any commercial or financial relationships that could be construed as a potential conflict of interest.
